# Strengthening national vaccine decision-making: Assessing the impact of SIVAC Initiative support on national immunisation technical advisory group (NITAG) functionality in 77 low and middle-income countries

**DOI:** 10.1016/j.vaccine.2018.11.070

**Published:** 2019-01-14

**Authors:** Kevin van Zandvoort, Natasha Howard, Sandra Mounier-Jack, Mark Jit

**Affiliations:** a Department of Infectious Disease Epidemiology, Faculty of Epidemiology and Population Health, London School of Hygiene & Tropical Medicine, Keppel Street, WC1E 7HT London, United Kingdom; b Department of Global Health and Development, Faculty of Public Health and Policy, London School of Hygiene & Tropical Medicine, 14-17 Tavistock Place, WC1H 9SH London, United Kingdom; c Modelling and Economics Unit, Public Health England, 61 Colindale Avenue, NW9 5EQ London, United Kingdom

**Keywords:** NITAG, SIVAC, Low and middle-income countries, Vaccination, Vaccine policy

## Abstract

**Background:**

National Immunisation Technical Advisory Groups (NITAGs) are multi-disciplinary expert groups that provide policy-makers with independent, evidence-based advice on vaccination. Between 2008 and 2017, the SIVAC Initiative supported establishment and strengthening of NITAGs in low and lower-middle income countries though its impact was never assessed quantitatively.

**Aim:**

To quantitatively assess whether SIVAC support is associated with a faster rate at which NITAGs became functional based on six performance indicators.

**Methods:**

Data from the World Health Organization/Unicef Joint Reporting Form (JRF) from 77 low and lower-middle-income countries were used to examine the time delay between the start of SIVAC support and NITAG functionality using a Cox proportional hazards model.

**Results:**

Countries receiving SIVAC support took a mean of 2.00 (95% CI 1.40–2.60) years to reported functionality compared to 2.82 (95% CI 2.05–3.59) years for countries without SIVAC support. We found evidence that SIVAC support is associated with reduced time until NITAG functionality, and this association cannot fully be explained by GDP per capita, percentage of GDP spent on healthcare, or NITAG functionality score at the start of the study period. However, quality of JRF data for the questions used to calculate NITAG functionality were poor, particularly for countries not receiving SIVAC support.

**Conclusion:**

SIVAC support is likely to have enabled many countries to more rapidly achieve NITAG functionality.

## 1 Introduction

Since the introduction of the Expanded Programme on Immunization in 1974, numerous vaccines have been developed against life-threatening diseases such as childhood diarrhoea, pneumonia, meningitis and cervical cancer. Accelerating the introduction of these vaccines can save lives, particularly in low- and middle-income countries (LMICs) [1]. However, navigating between multiple vaccine antigens, formulations, strategies and financing mechanisms requires careful appraisal of immunological, epidemiological, economic, logistic and sociological considerations. To support evidence-based decision making, the World Health Organization (WHO)'s Global Vaccine Action Plan 2011–2020 calls for all countries to establish or have access to a National Immunisation Technical Advisory Group (NITAG). NITAGs are multidisciplinary expert groups that can provide policy-makers with independent, evidence-based advice on vaccination [2].

From 2008 to 2017, the Bill & Melinda Gates Foundation funded the Supporting Independent Immunization and Vaccine Advisory Committees (SIVAC) Initiative. Hosted by Agence de Médecine Préventive, SIVAC supported the establishment and strengthening of NITAGs in low and middle-income countries [3]. While SIVAC funding ended in August 2017, the impact of its work over the past decade has not been quantitatively assessed. Qualitative research indicated that well-functioning NITAGs enhanced national immunisation programme strength and sustainability through providing country-owned evidence-based immunisation decision-making [4], [5]. Hence the aim of our study was to conduct the first quantitative assessment of the extent to which SIVAC support expedited the time to which NITAGs became functional. We focused on 77 countries eligible for support from Gavi, the Vaccine Alliance, in 2008, since SIVAC was created with a focus on Gavi-eligible countries.

## 2 Material and methods

### 2.1 NITAG functionality

Since 2010, the World Health Organization (WHO) and UNICEF have requested countries to self-report on six NITAG performance indicators in their annual Joint Reporting Form (JRF) [6]. These are listed in Box 1. We obtained responses for these indicators for the 2010–2016 JRFs, for 77 low-income and lower-middle-income countries that were Gavi-eligible in 2008. Responses were used to determine whether a NITAG had become functional depending on the number of questions that were answered with “Yes”. Different answers (i.e. “No”, “Not relevant”, missing values) did not contribute to the score.

Box 1 **NITAG performance indicators in the WHO/UNICEF Joint Reporting Form.**

A. Has the advisory group formal written Terms of References?
B. Are there legislative or administrative basis for the advisory group?
C. Are members of at least five of the following expertise areas represented in the group?
  1. Paediatricians;
  2. Public health experts;
  3. Infectious disease experts;
  4. Epidemiology experts;
  5. Immunology experts;
  6. Other experts;
D. Does the advisory group meet at least once per annum?
E. Are the agenda and background documents distributed (at least 1 week) prior to the meetings?
F. Are members of the advisory group required to disclose conflict of interest?

We defined a NITAG as being functional if all six criteria were answered with “yes”. As there were many missing data in the responses, we also considered an alternative where we restricted the definition to three key criteria, namely B, C, and D, indicating a legally-mandated, active NITAG with sufficiently broad membership. We further restricted this so criterion C required only four rather than at least five expertise areas. We refer to the first definition as the global definition and the latter as the restricted definition. In a sensitivity analysis, we explored the effect of using alternate definitions for NITAG functionality. NITAG functionality was defined as the year in which the NITAG first became functional (based on the chosen definition), regardless of whether the score subsequently decreased.

### 2.2 SIVAC support

We analysed data from 77 countries eligible for Gavi support in 2008. Out of these countries, using internal SIVAC documents, 2016 JRF data, and Gavi annual reports, we identified countries that were supported by SIVAC, at least peripherally, at some point between 2008 and 2017. Countries receive SIVAC support through a process in which countries are either (i) self-selected by requesting support and/or (ii) approached by SIVAC based on their assessment of the country. To estimate support level, we used a simple summative scoring system of reported SIVAC activities to categorise the 40 Gavi-eligible countries that SIVAC reported had received some level of support. First, each support activity was assigned a weighting from 1 to 4 based on its potential value in NITAG establishment or strengthening. For example, initial contact, first meeting participation, or introductory training were each assigned ‘1’. Similar follow-up activities (further meetings and training participation) were each assigned ‘2’. Finally, more involved activities that were crucial for establishment/strengthening, i.e. legal establishment and preparing a work-plan, were each assigned ‘4’. No ‘3’s were assigned, to maximise differentiation between countries receiving primarily follow-up versus crucial support activities. Total scores ranged between 2 and 19, and were divided into terciles at 10.00 and 2.67, giving high support (10.01–19.00), medium support (2.68–10.00), and low support (<2.68). As scoring was only intended as a rough guide, support terciles were then checked with SIVAC staff to determine appropriateness and identify anomalies. Countries classified as having low SIVAC support, but with a support score of 0, were considered to have received no SIVAC support. This reclassification led to a total of 31 Gavi-eligible countries receiving SIVAC support.

### 2.3 Statistical analyses

We performed descriptive analyses and Chi-squared tests to see whether there was a crude association between receiving SIVAC support and NITAG functionality. Moreover, we used Cox-proportional hazard models to investigate how SIVAC support affected time to NITAG functionality (see Supplemental File 2 for the model specification). Countries were included at the first year of the study period, and censored with an event in the year their NITAG became functional. As most countries received SIVAC support after the study period started, SIVAC support was modelled as a time-dependent variable. Hence, NITAG-years until receipt of SIVAC support contribute to the unsupported stratum whilst NITAG-years since receipt of SIVAC support contribute to the supported stratum, in order to prevent introduction of immortal time bias [7]. Similarly, as we are modelling SIVAC support as a time-dependent variable, we used extended Kaplan-Meier plots to graphically show rates over time [8].

We assumed that a country must have had at least one year of SIVAC support for support to have had an effect on time to NITAG functionality. For instance, SIVAC support in India began in 2013, but its NITAG also became functional in 2013. Thus, India did not contribute any NITAG-years to the SIVAC supported stratum in our survival analyses. Crude rate ratios were adjusted for gross domestic product (GDP) per capita, proportion of GDP spent on healthcare, and NITAG functionality score at the start of the study period. The first two indicators were obtained from the World Bank’s World Development Indicators for 2015 (http://wdi.worldbank.org).

### 2.4 Sensitivity analyses

Apart from presenting results for the global and restricted definitions of NITAG functionality, we assessed the sensitivity of our results against alternative definitions of NITAG functionality, namely scoring at least one, two, three, four, or five points on the six criteria mentioned above. We ran an additional analysis on a subset of the sample, limiting the sample to those Gavi-eligible countries (52 out of 77) that reported having a NITAG in JRF 2016. We did not use this sample in our primary analysis, as it significantly reduced the statistical power to detect meaningful effects. Finally, we assessed the effect of actual SIVAC support categories, rather than the dichotomous variable of receiving any SIVAC support. Again, this was not done in our primary analysis, as the number of countries in some of the support categories was very limited.

## 3 Results

### 3.1 Descriptive analyses

Table 1 shows that of 77 included countries, 31 received SIVAC support and 42 (50) achieved NITAG functionality in 2010–2016 when using the global (restricted) definition. There is some evidence that NITAG functionality is associated with receiving SIVAC support (χ^2^ = 4.59, p = 0.032) when using the global definition, and strong evidence (χ^2^ = 9.62, p = 0.002) when using the restricted definition of NITAG functionality. The year in which each individual country’s NITAG became functional (under multiple definitions of NITAG functionality) and periods in which countries received SIVAC support, is presented in Supplemental File 1.

**Table 1:** Number of NITAGs reported as functional in countries eligible for Gavi support in 2008, and mean number of years until functionality, by SIVAC support status.

Definition	Without SIVAC support		With SIVAC support	
	Total^i^	Mean (95%CI)	Total^i^	Mean (95%CI)
	*Based on countries achieving NITAG functionality in 2010*–*2016*			
Global	20/20 (100%)	2.82 (2.05–3.59)	22/22 (100%)	2.00 (1.40–2.60)
Restricted	23/23 (100%)	3.04 (2.35–3.74)	27/27 (100%)	1.88 (1.05–2.70)

	*Based on all countries*^ii^			
Global	20/46 (43.5%)	4.36 (3.90–4.82)	22/31 (71.0%)	2.16 (1.57–2.74)
Restricted	23/46 (50%)	4.30 (3.82–4.79)	27/31 (87.1%)	2.00 (1.46–2.54)

i Number of countries with a functional NITAG/Total number of countries included.

ii Assuming NITAGs become functional in the first possible year after the study period (i.e. 2017), thereby providing a lower bound on the number of years to functionality.

Many countries did not answer some or all of the questions about NITAG functionality. The missing data does not appear to be random, as SIVAC-supported countries are more likely to have complete data compared to unsupported countries, especially in later years. In 2010, the percentage of missing answers was 58% in countries without SIVAC support, compared to 44% in countries with SIVAC support (χ^2^ = 15.37, p < 0.001). In 2013, this became 47% compared to 32% (χ^2^ = 19.41, p < 0.001). In 2016, this became 49% compared to 10% (χ^2^ = 138.64, p < 0.001). Data for each year is presented in Supplemental File 1.

### 3.2 Time to NITAG functionality

Table 1 shows the average number of years until a country’s NITAG becomes functional, stratified by SIVAC support. Since 2010, restricting to NITAGs that achieved NITAG functionality in 2010–2016, it took a mean 2.82 (95%CI 2.05–3.59) years to become functional without SIVAC support, compared to 2.00 (95%CI 1.40–2.60) years with SIVAC support. Similarly, when assuming that countries that did not become functional would have become functional at the next possible year (2017), it took on average at least 4.36 (95%CI 3.90–4.82) years to become functional in the absence of SIVAC support, compared to 2.16 (95%CI 1.57–2.74) years with SIVAC support.

### 3.3 Regression analysis

**Fig. 1:**
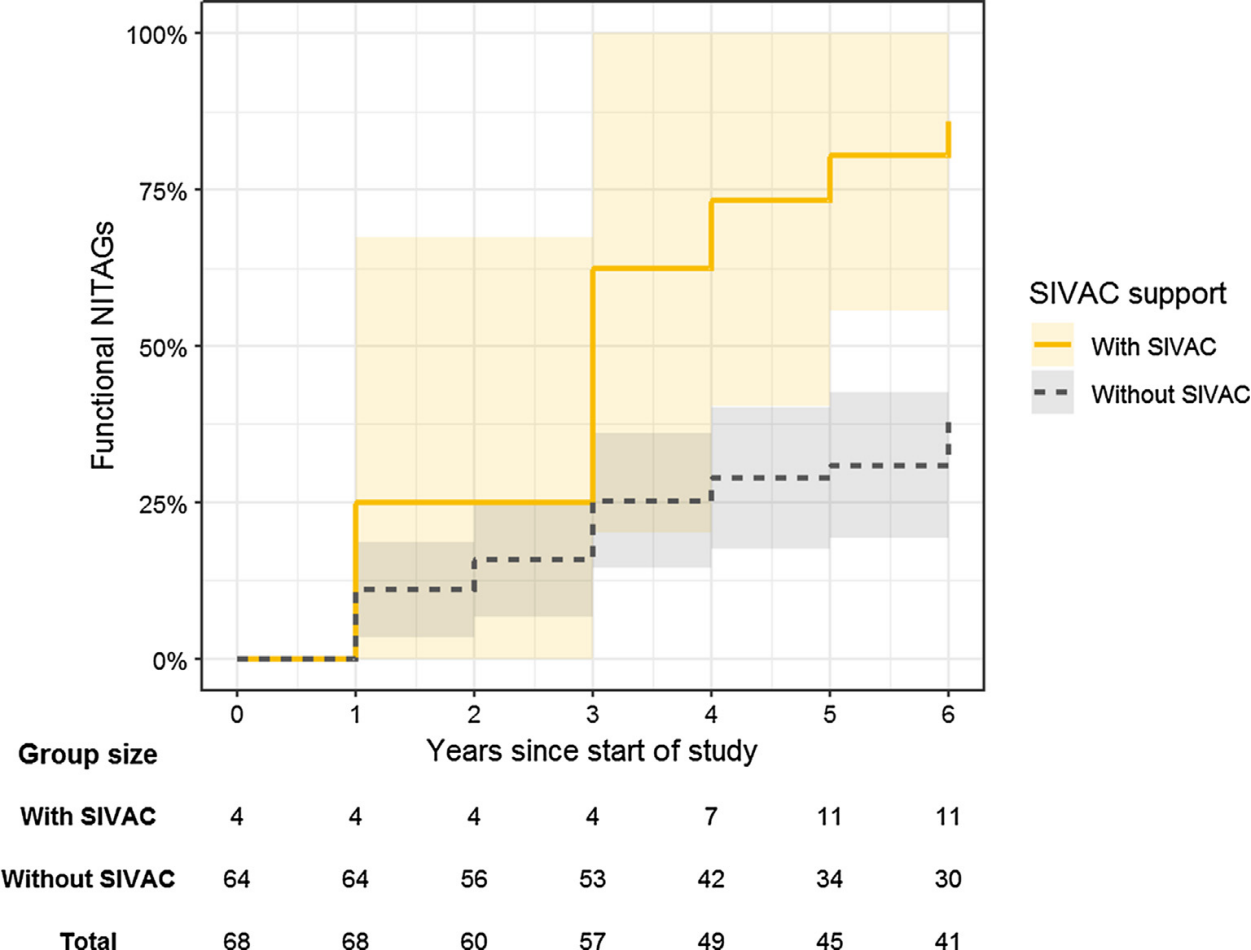
NITAG functionality by SIVAC support status. Extended Kaplan-Meier survival plot, showing failure curves of two hypothetical cohorts if SIVAC support status would not change throughout the follow-up period. Here, failure relates to the proportion of NITAGs that are functional under the global definition. The group size table shows the number of countries included within each stratum at each time point of the analysis.

Extended Kaplan-Meier survival estimates are presented in Fig. 1. SIVAC supported NITAGs seem to have become functional at a faster rate compared to NITAGs without SIVAC support. Estimated rates and rate ratios are presented in Table 2. There was strong evidence that the rate at which NITAGs became functional was higher for countries with SIVAC support (RR 3.68, 95%CI 1.70–7.95, p = 0.001). After adjusting for GDP per capita, percentage of GDP spent on healthcare, and NITAG functionality score in 2010, there remains good evidence that NITAGs in SIVAC supported countries became functional at a faster rate compared to NITAGs in countries without SIVAC support (RR 2.65, 95%CI 1.16–6.05, p = 0.021).

**Table 2:** Average rate, and crude and adjusted rate ratios for the association between receipt of SIVAC support and rate of NITAG functionality in countries eligible for Gavi support in 2008. Figures adjusted for GDP per capita, percentage of GDP spent on healthcare and SIVAC support score in 2010. p-values are derived from Wald-tests, and relate to the adjusted rate-ratios reported.

Definition	Stratum	Rate (95%CI)	RR (95%CI)	Adjusted RR (95%CI)	P-value
Global	Without SIVAC	0.08 (0.05–0.12)	3.68 (1.70–7.95)	2.65 (1.16–6.05)	0.021
	With SIVAC	0.27 (0.15–0.48)			

Restricted	Without SIVAC	0.10 (0.06–0.14)	3.53 (1.49–8.36)	2.65 (1.37–8.93)	0.009
	With SIVAC	0.33 (0.17–0.67)			

### 3.4 Sensitivity analyses

Results of all sensitivity analyses are presented in Supplemental File 3. Regardless of how NITAG functionality was defined, there was good evidence that the rate at which SIVAC supported NITAGs became functional was considerably higher compared to NITAGs without SIVAC support.

In one sensitivity analysis, we restricted our sample size to the 52 countries that reported having a functional NITAG in JFR 2016, i.e. excluding 25 previously included countries, of which 1 received SIVAC support. Note that 11 of these excluded countries actually did report having a NITAG at least once prior to 2016. We find similar values to the primary analysis for the mean number of years it takes until NITAGs become functional in each group. However, the average rate at which NITAGs without SIVAC support become functional is slightly higher, and crude and adjusted rate ratios become smaller (RR 1.90, 95%CI 0.83–4.34, p = 0.127; adjusted RR 1.77, 95%CI 0.73–4.27, p = 0.207). Using this restricted sample size, there is no longer any statistical evidence of a true effect.

When modelling the effect of different SIVAC support categories (low support; medium support; high support), we only find evidence that NITAGs receiving high SIVAC support became functional at a faster rate compared to NITAGs without any SIVAC support (adjusted RR 6.14, 95%CI 2.20–17.15, p-value 0.001). However, it should be noted that sample sizes in individual SIVAC support categories were very low.

## 4 Discussion

Our analysis shows that SIVAC support significantly reduced the time to NITAG functionality, as defined by JRF criteria. The estimated mean delay between start of SIVAC support and NITAG functionality, as estimated using either the global or restricted definition, is around 2 years, almost a year shorter compared to unsupported countries. Similarly, the rate at which NITAGs became functional was around 3.5 times faster in countries receiving SIVAC support. Although that association could be partly explained by GDP per capita, percentage of GDP spent on healthcare, and SIVAC support score in 2010, the estimated rate remained around 2.5 times higher after adjusting for these covariates. These results hold when using less stringent definitions of NITAG functionality, as shown in Supplemental File 3. Supported NITAGs consistently become functional at a faster rate compared to NITAGs in countries without SIVAC support.

While SIVAC support was aimed at both development and strengthening of NITAGs, we only assessed the latter. Although NITAG development is crucial, only functional NITAGs are likely to be significant partners in national public health policy-making processes and we chose to focus on functionality as the most meaningful outcome. However, evaluation of SIVAC’s full contribution would also include NITAG establishment. Similarly, we did not assess cost-effectiveness, as SIVAC support was tailored based on country needs assessment and thus highly contextual (e.g. some countries cost more to support due to being more decentralised, requiring more technical training) and comparing countries was not always meaningful.

Including countries without a NITAG may impose a selection bias, as a country needs to have a NITAG in order to become functional. Therefore, we limited our sample to only those countries reporting a NITAG in JRF 2016 in our sensitivity analysis, decreasing the sample size from 77 to 52 countries. Although our model still estimates an increased rate of NITAG functionality in SIVAC supported countries, its confidence interval is too wide and P-value too large to rule out a chance finding. By limiting our sample size, we decrease our statistical power to detect an effect. Moreover, of the 25 countries excluded, 11 reported having a NITAG in one or more of the JRFs prior to 2016. Therefore, we are unsure how reliable this answer is to measure NITAG availability in 2016.

This analysis is consistent with qualitative research showing that SIVAC-supported countries exhibited satisfactory levels of functionality e.g. underpinned by clear standard operating procedures, adequate member specialisation, conflict of interest policy, annual workplans, operational working groups, recommendations submitted to the Ministry of Health [4], [9]. The SIVAC model has been characterised as a step-by-step approach to establish and/or strengthen NITAGs, through a combination of tools and technical support tailored to the local context [3], [10]. SIVAC support often involved direct financing, which though limited in value was often viewed by NITAGs as catalytic for development. With the ending of SIVAC and likely fragmentation of institutional support to NITAGs in LMICs, there is a risk that functionality and further progress will stall or even reverse [5]. Beyond fulfilling JRF functionality criteria, NITAGs in Gavi-eligible countries remain fragile and many will need ongoing strengthening before they can embed themselves sustainably within national decision-making processes. A more meaningful way than JRF criteria for assessing functionality might be evaluation of NITAG process and output quality (e.g., national adoption of vaccine recommendations, long-term NITAG inputs into national immunisation programme strategies). For example, a key difference between well-functioning and poorly-functioning NITAGs was the capacity to consider cost-effectiveness and funding sustainability within vaccine recommendations [4], [5], [11].

### 4.1 Limitations

There are several limitations to this analysis. First, questions about NITAGs in Joint Reporting Forms were self-reported and not verified by independent monitors. There was a considerable amount of missing answers to multiple questions used to compute NITAG functionality scores, and there was evidence that missing data were more pronounced for countries without SIVAC-support than countries with SIVAC support. This may underestimate the number of countries achieving NITAG functionality without SIVAC support, especially when more stringent definitions for NITAG functionality are used. We used alternative definitions for NITAG functionality to partly overcome this issue. Moreover, the reason that data quality improved for SIVAC supported countries may be itself an indicator that functionality of those NITAGs improved.

Second, we assumed that NITAGs became functional in the first year in which the required NITAG functionality score for a given definition was met. In some countries, NITAG functionality scores subsequently decreased, which might be a result of missing data, but may also reflect that NITAG functionality was not sustainable.

Third, the study period (2010–2016) for which data were available was relatively short. A longer period, or more detailed data, may have given a better view for the exact time-delay for countries that did not achieve NITAG functionality in this analysis.

Fourth, sample sizes in specific SIVAC-support categories were low, resulting in large confidence intervals. Irrespective of statistical power, point estimates in each support category still pointed to a signal of increased rates in all SIVAC-support categories. There does not seem to be sufficient statistical power to investigate the effect of individual support categories.

Fifth, countries were predominantly self-selected, rather than being randomly assigned whether they received SIVAC support or not. This may impose a selection bias, as countries receiving technical support may have been more likely to develop a functional NITAG even in the absence of SIVAC support. Countries receiving SIVAC support were indeed more likely to have answered the NITAG-performance indicators mentioned in box 1, even prior to receipt of SIVAC support. We aimed to mitigate this effect in our analysis, by adjusting for countries’ NITAG functionality scores at the start of the study period in our analyses.

Finally, while rate ratios were adjusted for GDP per capita, percentage of GDP spent on healthcare, and NITAG functionality score at the start of the study period, the observed effects may have been subject to residual confounding by factors that were beyond the scope of the study to quantify, such as political climate or immunisation programme functionality.

## 5 Conclusions

Overall, SIVAC support significantly accelerated the time to functionality for NITAGs. Functional NITAGs are important, as they provide independent evidence-informed decision-making for national immunisation programmes. Despite the success of SIVAC support, 25 of the 77 countries included in this analysis still did not report being functional in 2016, according to JRF criteria. Given the successes of SIVAC, a successor may help those countries establish functional NITAGs. Moreover, NITAG functionality should be regularly monitored, to determine whether NITAGs remain functional now SIVAC support has ended.

## Funding

The Bill & Melinda Gates Foundation (BMGF) provided study funding (grant IID46303). Views expressed are those of the authors and not necessarily reflective of the views of LSHTM or BMGF.

## Conflicts of interest

The authors declare that they have no conflicts of interest.

## References

[1] Lee L.A., Franzel L., Atwell J. (2013). The estimated mortality impact of vaccinations forecast to be administered during 2011-2020 in 73 countries supported by the GAVI Alliance. Vaccine.

[2] Duclos P. (2010). National Immunization Technical Advisory Groups (NITAGs): guidance for their establishment and strengthening. Vaccine.

[3] Adjagba A., Senouci K., Biellik R. (2015). Supporting countries in establishing and strengthening NITAGs: lessons learned from 5 years of the SIVAC initiative. Vaccine.

[4] Howard N., Walls H., Bell S., Mounier-Jack S. (2018). The role of National Immunisation Technical Advisory Groups (NITAGs) in strengthening national vaccine decision-making: a comparative case study of Armenia, Ghana, Indonesia, Nigeria, Senegal and Uganda. Vaccine.

[5] Howard N., Bell S., Walls H. (2018). The need for sustainability and alignment of future support for National Immunization Technical Advisory Groups (NITAGs) in low and middle-income countries. Hum Vaccines Immunother.

[6] Duclos P., Ortynsky S., Abeysinghe N. (2012). Monitoring of progress in the establishment and strengthening of national immunization technical advisory groups. Vaccine.

[7] Suissa S. (2008). Immortal time bias in pharmaco-epidemiology. Am J Epidemiol.

[8] Snapinn S.M., Jiang Q., Iglewicz B. (2005). Illustrating the impact of a time-varying covariate with an extended Kaplan-Meier estimator. Am Stat.

[9] Ba-Nguz A., Adjagba A., Wisnu Hendrarto T., Sewankambo N.K., Nalwadda C., Kisakye A. (2017). The role of national immunization technical advisory groups (NITAGs) in the introduction of inactivated polio vaccine: experience of the Indonesia and Uganda NITAGs. J Infect Dis.

[10] Senouci K., Blau J., Nyambat B. (2010). The Supporting Independent Immunization and Vaccine Advisory Committees (SIVAC) initiative: a country-driven, multi-partner program to support evidence-based decision making. Vaccine.

[11] Bell S, Mounier-Jack S, Blanchard L, Walls H, Howard N. Value and effectiveness of National Immunization Technical Advisory Groups (NITAGs) in low and middle-income countries: a qualitative study of global and national perspectives; 2018 [unpublished]. 10.1093/heapol/czz027 PMC6661538 31074778

