# Supplementary material for: Strengthening national vaccine decision-making: Assessing the impact of SIVAC Initiative support on national immunisation technical advisory group (NITAG) functionality in 77 low and middle-income countries
Source: Vaccine. 2019 Jan 14;37(3):430–4. doi: 10.1016/j.vaccine.2018.11.070 (PMC6334253; doi:10.1016/j.vaccine.2018.11.070)
Supplement: Supplementary data 1 [file mmc1.docx]

**Supplemental File 1: List of countries included, year at which NITAGs became functional, year at which SIVAC support started, and completeness for each question**

*
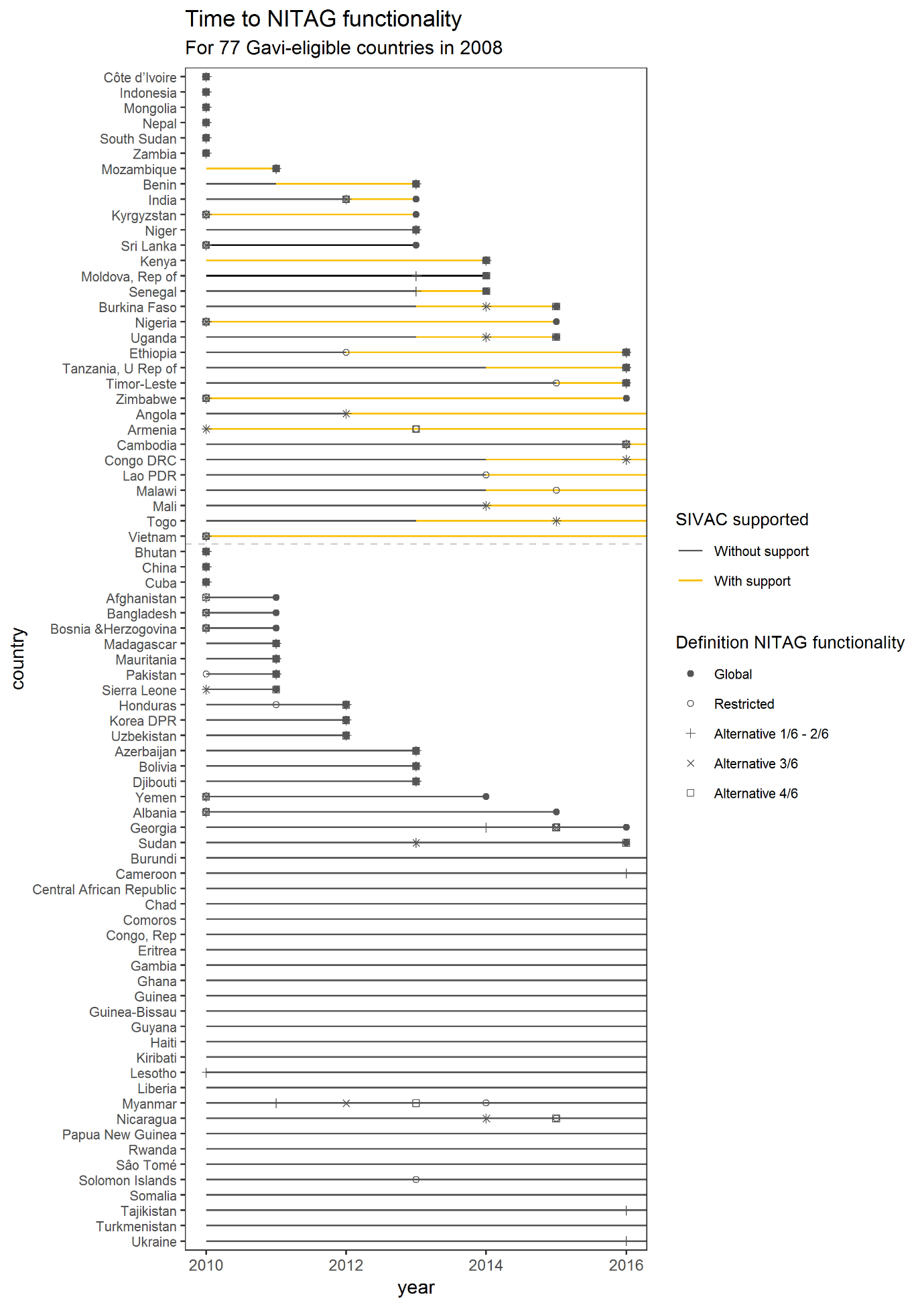
*

Figure 1. Time line plot showing NITAG functionality and SIVAC support across calendar time for 77 Gavi2008-eligible countries. Yellow segments indicate periods in which countries received SIVAC support. Solid points indicate year where a NITAG reached functionality according to the global definitions, whilst other shapes indicate NITAG functionality according to alternative shapes. Shapes may overlap, indicating that using an alternative definition of NITAG functionality did not affect year at which the NITAG was determined to be functional.

Table 1. Percentage of questions with missing answers, by SIVAC support and year.

| **Year** | **Without SIVAC** | **With SIVAC** | **χ^2^** | **P-value** |
| --- | --- | --- | --- | --- |
| 2010 | 57.71% | 43.99% | 15.37 | <0.001 |
| 2011 | 49.01% | 47.21% | 0.26 | 0.608 |
| 2012 | 47.83% | 41.64% | 3.14 | 0.076 |
| 2013 | 46.84% | 31.67% | 19.41 | <0.001 |
| 2014 | 42.29% | 25.81% | 24.13 | <0.001 |
| 2015 | 48.42% | 18.18% | 80.60 | <0.001 |
| 2016 | 49.21% | 10.26% | 138.64 | <0.001 |
