# Supplementary material for: Strengthening national vaccine decision-making: Assessing the impact of SIVAC Initiative support on national immunisation technical advisory group (NITAG) functionality in 77 low and middle-income countries
Source: Vaccine. 2019 Jan 14;37(3):430–4. doi: 10.1016/j.vaccine.2018.11.070 (PMC6334253; doi:10.1016/j.vaccine.2018.11.070)
Supplement: Supplementary data 2 [file mmc2.docx]

**Supplemental File 2. Specification of the Cox proportional hazard model used in the analysis.**

Survival analyses were performed in STATA15. We will here present the models underlying these analyses.

The model for the crude rate ratios can be written as:

| $h\left( t \right)=h_{0}(t)e^{\beta_{1}X_{1}(t)}$ | (1) |
| --- | --- |

where *X_1_(t)* indicates the value of the covariate. SIVAC support was included as a time varying variable, where *X_1_(t)* = 0 where *t* is smaller than the year at which SIVAC support was started, and *X_1_(t)* = 1 when *t* is equal to or larger than the year at which SIVAC support was started. *β_1_* is it’s associated coefficient, and *h_0_* is the basic rate when *X_1_* is 0. Crude rate ratios (HR) can then be calculated as:

| $HR=\frac{h(t)}{h_{0}(t)}=e^{\beta_{1}}$ | (2) |
| --- | --- |

The adjusted model can be written as:

| $h\left( t \right)=h_{0}(t)e^{\beta_{1}X_{1}(t)+\beta_{2}X_{2}+\beta_{3}X_{3}+\beta_{3}X_{3}}$ | (3) |
| --- | --- |

Where *X_1_(t)* and *β_1_* have the same meaning as before. *X_2_-X_4_* are country specific values for GDP per capita of the country, percentage of GDP spent on healthcare, and NITAG functionality score at the start of the study period. *β_2_- β_4_* are their associated coefficients, and *h_0_* is the baseline rate when *X_1_-X­_3_* are all 0.
