# Supplementary material for: Strengthening national vaccine decision-making: Assessing the impact of SIVAC Initiative support on national immunisation technical advisory group (NITAG) functionality in 77 low and middle-income countries
Source: Vaccine. 2019 Jan 14;37(3):430–4. doi: 10.1016/j.vaccine.2018.11.070 (PMC6334253; doi:10.1016/j.vaccine.2018.11.070)
Supplement: Supplementary data 3 [file mmc3.docx]

**Supplemental File 3. Sensitivity analyses**

This file shows the results of the three additional sensitivity analyses ran: 1) using alternative definitions for NITAG functionality; 2) restricting the sample size to 52 countries reporting having a NITAG in JRF 2016; and 3) assessing the effect of level of SIVAC support received.

*1. Alternative definitions*

To test the sensitivity of the definition of NITAG functionality, we ran the analyses using alternative definitions. NITAG functionality was redefined as scoring at least one, two, three, four, or five out of the six criteria mentioned before. Results of these analyses are presented in tables 1 – 4. Definitions for scoring at least 1/6 and 2/6 are combined as these compare the exact same countries, achieving functionality at the exact same times (see figure A1.1). Similarly, the definition for scoring at least 5/6 is omitted from these tables as this resulted in exactly the same countries achieving functionality at the exact same times as in the global definition in the main text (which in essence is scoring 6/6).

Although the number of NITAGs that became functional is slightly affected by the definition used to determine NITAG functionality, there remains good evidence that SIVAC support is positively associated with NITAG functionality, as shown in table 1.

Table 1. Number of NITAGs classified as functional.

| **Definition** | **Functional without SIVAC** | **Functional with SIVAC** | **χ^2^** | **P-value** |
| --- | --- | --- | --- | --- |
| Alternative 1/6 – 2/6 | 26/46 | 29/31 | 10.69 | 0.001 |
| Alternative 3/6 | 22/46 | 29/31 | 15.33 | <0.001 |
| Alternative 4/6 | 22/46 | 25/31 | 7.06 | 0.008 |

The mean number of years it takes a NITAG become fully functional is similar between definitions, it remains around three years for countries without SIVAC support and two years for countries with SIVAC support, although estimates slightly increase when more stringent definitions are used. When also including countries which don’t achieve NITAG functionality status throughout the study period, the mean number of years it will at least take for a NITAG to become functional is around 4.2 years for those without SIVAC support and two years for those with SIVAC support. There are no large differences between definitions.

Table 2. Mean number of years until NITAGs become functional.

| **Definition** | **Without SIVAC support** | | **With SIVAC support** | |
| --- | --- | --- | --- | --- |
|  | Mean | 95%CI | Mean | 95%CI |
| *Based on countries achieving NITAG functionality in 2010-2016* | | | | |
| Alternative 1/6 – 2/6 | 3.09 | 2.37-3.80 | 1.8 | 1.14-2.46 |
| Alternative 3/6 | 2.79 | 2.14-3.44 | 1.73 | 1.12-2.33 |
| Alternative 4/6 | 2.95 | 2.17-3.73 | 1.89 | 1.18-2.60 |
| *Based on all countries* | | | | |
| Alternative 1/6 – 2/6 | 4.28 | 3.79-4.78 | 1.75 | 1.20-2.30 |
| Alternative 3/6 | 4.37 | 3.89-4.85 | 1.69 | 1.18-2.21 |
| Alternative 4/6 | 4.41 | 3.94-4.88 | 1.93 | 1.49-2.38 |

As shown in tables 3 and 4, there is strong evidence across all definitions that SIVAC support is associated with an increased rate of NITAG functionality. This effect remains when adjusting for potential confounding factors.

Table 3. Crude rate ratios showing association between receiving SIVAC support and the rate of a NITAG becoming functional across alternative definitions of NITAG functionality.

| **Definition** | **Stratum** | **Rate** | **95% CI** | **Rate ratio** | **95%CI** | **P-value** |
| --- | --- | --- | --- | --- | --- | --- |
| Alternative 1/6 – 2/6 | without SIVAC | 0.10 | 0.07-0.15 | 4.71 | 2.14-10.40 | <0.001 |
|  | with SIVAC | 0.48 | 0.26-0.89 |  |  |  |
| Alternative 3/6 | without SIVAC | 0.08 | 0.05-0.13 | 6.64 | 3.01-14.65 | <0.001 |
|  | with SIVAC | 0.50 | 0.28-0.90 |  |  |  |
| Alternative 4/6 | without SIVAC | 0.08 | 0.05-0.12 | 4.60 | 1.90-11.10 | 0.001 |
|  | with SIVAC | 0.31 | 0.16-0.60 |  |  |  |

Table 4. Adjusted rate ratios showing association between receiving SIVAC support and the rate of a NITAG becoming functional across alternative definitions of NITAG functionality.

| **Definition** | **Rate ratio** | **95% CI** | **P-value** |
| --- | --- | --- | --- |
| Alternative 1/6 – 2/6 | 4.93^i^ | 2.04-11.88 | <0.001 |
| Alternative 3/6 | 7.05 ^i^ | 2.86-17.35 | <0.001 |
| Alternative 4/6 | 4.89 ^i^ | 1.79-13.34 | 0.002 |
| i. Adjusted for GDP per capita, percentage of GDP spent on healthcare, and NITAG functionality score at the start of the study period. | | | |

*2. Alternative sample size*

Tables 5, 6, and 7 show the results of our analysis when only including countries that reported having a NITAG in JRF 2016. This reduced the sample size from 77 to 52 countries, subsequently reducing the statistical power.

Table 5. Mean number of years until NITAGs becomes functional.

| **Definition** | **Without SIVAC support** | | **With SIVAC support** | |
| --- | --- | --- | --- | --- |
|  | Mean | 95%CI | Mean | 95%CI |
| *Based on countries achieving NITAG functionality in 2010-2016* | | | | |
| Global | 3 | 2.15-3.85 | 2 | 1.40-2.60 |
| Restricted | 3.26 | 2.48-4.05 | 1.88 | 1.05-2.70 |
| *Based on all countries* | | | | |
| Global | 3.69 | 3.14-4.25 | 2.17 | 1.55-2.79 |
| Restricted | 3.52 | 2.93-4.10 | 2 | 1.40-2.60 |

Table 6. Crude rate ratios showing association between receiving SIVAC support and the rate of a NITAG becoming functional compared to receiving no SIVAC support.

| **Definition** | **Stratum** | **Rate** | **95% CI** | **Rate ratio** | **95%CI** | **P-value** |
| --- | --- | --- | --- | --- | --- | --- |
| Global | without SIVAC | 0.13 | 0.08-0.21 | 1.90 | 0.83-4.34 | 0.127 |
|  | with SIVAC | 0.28 | 0.16-0.51 |  |  |  |
| Restricted | without SIVAC | 0.17 | 0.11-0.27 | 1.49 | 0.59-3.74 | 0.399 |
|  | with SIVAC | 0.36 | 0.18-0.73 |  |  |  |

Table 7. Adjusted rate ratios showing association between receiving SIVAC support and the rate of a NITAG becoming functional compared to receiving no SIVAC support.

| **Definition** | **Rate ratio** | **95% CI** | **P-value** |
| --- | --- | --- | --- |
| Global | 1.77^i^ | 0.73-4.27 | 0.207 |
| Restrictive | 1.71^i^ | 0.60-4.88 | 0.315 |
| i. Adjusted for GDP per capita, percentage of GDP spent on healthcare, and NITAG functionality score at the start of the study period. | | | |

*3. SIVAC support categories*

Table 8. Crude rate ratios showing association between receiving low, medium, or high SIVAC support and the rate of a NITAG becoming functional compared to receiving no SIVAC support.

| **Definition** | **Stratum** | **Rate** | **95% CI** | **Rate ratio** | **95%CI** | **P-value** |
| --- | --- | --- | --- | --- | --- | --- |
| Global | without SIVAC | 0.08 | 0.05-0.12 | 1 | . | . |
|  | low SIVAC | 0.25 | 0.04-1.77 | 2.92 | 0.35-24.56 | 0.325 |
|  | medium SIVAC | 0.19 | 0.06-0.58 | 2.45 | 0.39-8.76 | 0.167 |
|  | high SIVAC | 0.33 | 0.16-0.70 | 4.59 | 1.93-10.88 | 0.001 |
| Restricted | without SIVAC | 0.10 | 0.06-0.14 | 1 | . | . |
|  | low SIVAC | 0 | . | . | . | . |
|  | medium SIVAC | 0.29 | 0.07-1.14 | 3.01 | 0.64-14.20 | 0.164 |
|  | high SIVAC | 0.4 | 0.18-0.89 | 4.07 | 1.62-10.25 | 0.003 |

Table 9. Adjusted rate ratios showing association between receiving low, medium, or high SIVAC support and the rate of a NITAG becoming functional compared to receiving no SIVAC support.

| **Definition** | **Stratum** | **Rate ratio** | **95%CI** | **P-value** |
| --- | --- | --- | --- | --- |
| Global | without SIVAC | 1 | . | . |
|  | low SIVAC | 1.82^i^ | 0.19-17.10 | 0.598 |
|  | medium SIVAC | 1.21^i^ | 0.32-4.55 | 0.783 |
|  | high SIVAC | 4.39^i^ | 1.78-10.82 | 0.001 |
| Restricted | without SIVAC | 1 | . | . |
|  | low SIVAC | . ^ii^ | . | . |
|  | medium SIVAC | 1.65^i^ | 0.32-8.44 | 0.547 |
|  | high SIVAC | 6.14^i^ | 2.20-17.15 | 0.001 |
| i. Adjusted for GDP per capita, percentage of GDP spent on healthcare, and NITAG functionality score at the start of the study period. ii. Adjusted rates could not be computed for the low SIVAC support category due to data constraints. | | | | |
